# Digital Health Technologies in the Treatment of Chronic Pelvic Pain Syndromes: A Systematic Review of Randomized Clinical Trials

**DOI:** 10.3390/healthcare13212665

**Published:** 2025-10-22

**Authors:** Irene Torres-Sánchez, Olga Tejada-Vega, Guadalupe Rebollo-Segovia, Laura López-López, Esther Díaz-Mohedo

**Affiliations:** 1 Department of Physiotherapy, Faculty of Health Sciences, University of Granada, 18016 Granada, Spain; irenetorres@ugr.es; 2 Department of Physiotherapy, Faculty of Health Sciences, University of Malaga, 29071 Malaga, Spain; olga.tejada.v15@gmail.com (O.T.-V.); lupers2000@gmail.com (G.R.-S.); estherdiaz@uma.es (E.D.-M.)

**Keywords:** digital health, eHealth, mHealth, chronic pelvic pain, endometriosis, dysmenorrhea, interstitial cystitis, virtual reality, mobile applications

## Abstract

**Objective:** To comprehensively examine the qualitative results of current studies reporting the efficacy of digital health (DH) technologies in the treatment of chronic pelvic pain syndromes (CPPS) and to describe the characteristics of these interventions. **Materials and methods:** In line with Preferred Reporting Items for Systematic Reviews and Meta-Analyses guidelines, CINAHL, MEDLINE (via PubMed), Scopus and Web of Science databases were searched for trials published between database inception and July 2024. Randomized clinical trials using DH technologies for CPPS treatment were included. Methodological quality and risk of bias were appraised using the Downs and Black scale and the Cochrane Risk of Bias Assessment Tool. **Results:** Six articles were included. Four studies showed pain reduction in both groups between baseline and the end of the study, but this reduction was significant only in the experimental groups. One study showed an increase in pain intensity in all the groups, although it was smaller in the experimental groups. The last study found significant pain reduction in both groups, with no significant difference between them. **Conclusions:** DH technologies seem to offer some improvement in CPPS patients’ pain intensity. However, the studies showed high heterogeneity, which influences the consistency of the results.

## 1. Introduction

According to the European Association of Urology, chronic pelvic pain is defined as “chronic or persistent pain perceived in structures related to the pelvis of either men or women. It is often associated with negative cognitive, behavioral, sexual and emotional consequences as well as with symptoms suggestive of lower urinary tract, sexual, bowel, pelvic floor or gynecological dysfunction” [1]. It can be subdivided into “specific disease-associated pelvic pain” and “chronic pelvic pain syndrome”. Chronic pelvic pain syndrome is the occurrence of chronic pain when there is no proven infection or other obvious local pathology that may account for the pain. It encompasses a variety of diseases such as dysmenorrhea, chronic prostatitis and irritable bowel syndrome [2]. As it is a complex condition or range of conditions, there are no worldwide prevalence data for chronic pelvic pain syndromes. However, prevalence rates for pelvic pain conditions are generally higher for women than men. The quality of life of women is also affected to a greater extent than that of men [3].

The mechanism underlying chronic pain conditions is not entirely known, but it is thought to be related to central sensitization [2]. Evidence of central sensitization in women with urogynecological chronic pelvic pain has been supported by reviews [4,5], trials [6,7] and expert consensus [8]. Central pain in patients with chronic pelvic pain presents itself as a disproportionate, non-mechanical, non-anatomically distributed, unpredictable pattern of pain provocation in response to multiple/non-specific aggravating/easing factors. It persists beyond expected tissue exposure healing/pathology recovery times. Hyperalgesia, allodynia and/or hyperpathia are common clinical findings, as well as symptoms of autonomic nervous system dysfunction (e.g., skin discoloration, excessive sweating, trophic changes, dysesthesias). This high-severity pain exists in association with high levels of functional disability as well as psychosocial factors (e.g., catastrophizing, distress, poor self-efficacy, maladaptive beliefs and pain behaviors, altered family/work/social life, medical conflict) [9]. Furthermore, beyond its challenging clinical management, chronic pelvic pain imposes a substantial economic and social burden on individuals and healthcare systems. A systematic review of cost-of-illness studies in women with chronic pelvic pain reported that annual direct healthcare costs range roughly from USD 1367 to 7043 per woman, out-of-pocket expenditures range from USD 193 to 2457, and productivity losses (from absenteeism or reduced performance) range from USD 4216 to 12,789 per woman per year; total costs in some settings reached up to USD 20,898 annually [10].

Physical therapy plays an important role in conservative management of chronic pain [11]. Some recommended therapies are pelvic floor muscle training [12], pelvic floor relaxation techniques [13], manual therapy [14], acupuncture [15] and electrotherapies (e.g., extracorporeal shockwave therapy [16], transcutaneous electrical nerve stimulation [17] and posterior tibial nerve stimulation [18]). Cognitive interventions such as pain education [19], mindfulness [20] and mental imagery [21] should also be considered. As healthcare continues to evolve, new technologies have emerged as promising complements to traditional therapies.

Digital health is the field of knowledge and practice associated with the development and use of digital technologies to improve health [22]. It expands the concept of eHealth [23] to include digital consumers, with a wider range of smart devices and connected equipment. The term “digital health” is often used as a broad umbrella term encompassing eHealth, mHealth, digital medicine and digital therapeutics, as well as developing areas such as the use of advanced computing sciences [24].

According to the Food and Drug Administration, digital health technologies are intended to provide patients with personalized treatment and support for chronic diseases, including those that require continuous monitoring [25]. The Digital Therapeutics Alliance classifies these technologies into various intervention types such as behavioral interventions, cognitive-behavioral therapies, and digital therapeutics for chronic conditions like chronic pelvic pain [26]. These classifications are based on their intended use and target populations. For example, some digital therapeutics focus on managing chronic pain, while others promote behavioral changes that reduce symptoms or improve quality of life.

Numerous studies have shown that digital health technologies can improve health conditions. Examples include virtual reality for adults with chronic low back pain [27], internet-delivered cognitive and behavioral interventions for adults with chronic pain [28] and mobile health applications for the most prevalent conditions [29].

The rationale for conducting this systematic review stems from the growing interest and application of digital health technologies in managing chronic conditions, including Chronic Pelvic Pain Syndrome. Despite being promising, digital health technologies have faced significant challenges in treatment protocols, particularly concerning user adherence. Studies have highlighted a decline in intervention effectiveness over time due to poor engagement and adherence to digital interventions, which remains a barrier to long-term treatment success [30].

To our knowledge, no systematic reviews have explored the efficacy of digital health technologies in chronic pelvic pain syndromes. Therefore, the aim of this systematic review was to comprehensively examine the qualitative results of current studies reporting the efficacy of digital health technologies in the treatment of chronic pelvic pain syndromes and to describe the characteristics of these interventions.

## 2. Materials and Methods

### 2.1. Design

A systematic review of randomized clinical trials was conducted to evaluate the effectiveness of digital health technologies in physiotherapy for treating chronic pelvic pain syndromes. Preferred Reporting Items for Systematic Reviews and Meta-Analyses (PRISMA) [31] was used to carry out this review. The review was previously registered in the International Prospective Register of Systematic Reviews (PROSPERO) with number CRD42024540881. Available from: https://www.crd.york.ac.uk/prospero/display_record.php?RecordID=540881.

### 2.2. Search Strategy

The literature research was performed in four databases (CINAHL, MEDLINE (via PubMed), Scopus and Web of Science) from their inception up to July 2024, with no language restrictions. To identify additional relevant articles, we also examined the reference lists of other reviews and related publications.

Supplementary Material S1 provides the search strategy used in each database. It included terms related to “Chronic pelvic pain” and terms related to digital health using the Boolean operators “AND” and “OR”. The search strategy used in MEDLINE (via PubMed) was as follows: (“Pelvic Pain”[MeSH] OR “chronic pelvic pain”) AND (“prostate” OR “Prostatitis”[MeSH] OR “bladder” OR “scrotal” OR “testicular” OR “epididymal” OR “penile” OR “urethral” OR “post-vasectomy” OR “vulvar” OR “vestibular” OR “clitorial” OR “endometriosis” OR “CPPPS” OR “dysmenorrhea” OR “irritable bowel” OR “chronic anal” OR “intermittent chronic anal” OR “pudendal pain syndrome” OR “Dyspareunia”[MeSH] OR “sexual dysfunction” OR “pelvic organ” OR “pelvic floor muscle” OR “abdominal muscle” OR “spinal” OR “coccyx” OR “hip muscle” OR “chronic post-surgical pain syndrome”) AND (“Video Games”[MeSH] OR “Game*” OR “Gaming” OR “gamification” OR “Exergaming”[MeSH] OR “exergam*” OR “Wii” OR “Nintendo” OR “Kinect” OR “Xbox” OR “PlayStation” OR “virtual” OR “Virtual Reality”[MeSH] OR “Virtual Reality Exposure Therapy”[MeSH] OR “computer-based” OR “Steam” OR “Mobile Applications”[MeSH] OR “app” OR “app-based” OR “Digital Health”[MeSH] OR “mHealth” OR “eHealth” OR “Telemedicine”[MeSH] OR “technologies” OR “artificial intelligence” OR “telehealth” OR “biomedical technology”[MeSH] OR “medical informatics applications”[MeSH] OR “smartphone” OR “mobile*” OR “computer” OR “wearable devices”). The full search strategy for each database is available in Supplementary Material S1.

### 2.3. Study Selection

Articles were selected using the PICOS (Participants, Intervention, Comparisons, Outcomes and Study) question, which is commonly used in systematic reviews to define the key components of the research question [32]. The PICOS question consisted of: Population: Adults with any form of chronic pelvic pain syndromes; Intervention: digital health technologies for treatment; Comparisons: control group, no intervention, intervention without digital health, digital health intervention, placebo, traditional treatment; Outcomes: pain; Study Design: randomized clinical trials. The exclusion criteria for studies were texts not available in Spanish, English or French, and lack of access to the full text. To operationalize the PICOS framework, we developed a search strategy that reflected its components and reviewed articles that matched each component of the framework. The Population component was addressed using terms such as “chronic pelvic pain”, “pelvic pain syndrome”, “bladder pain syndrome”, and similar related conditions. For the Intervention, we included keywords such as “digital health”, “mobile health”, “telemedicine”, “eHealth”, “apps”, and “internet-based intervention”. The Comparator, the outcomes and the study design were not restricted during the search but were considered during the selection phase. Details of inclusion and exclusion criteria are shown in Table 1.

Once the results were obtained in each database, we excluded duplicate articles. A preliminary screening was performed by reading only the title and abstract of the articles to see if they met the inclusion criteria. Finally, a second screening was conducted on the remaining articles, reading the full text to check compliance with all criteria. The results of this second screening are shown in Supplementary Material S2. The analysis was conducted by two independent reviewers (OTV and GRV), and a third reviewer (ITS) helped to reach consensus in case of any disagreement.

### 2.4. Data Extraction

Two reviewers (OTV and GRV) carried out the data extraction. When the information was insufficient, we emailed the corresponding author for clarification. A third reviewer (ITS) was consulted in case of disagreements to reach consensus. The extracted data were organized into two summary tables. A table reports the characteristics of included studies: author, year, country, sample size, mean age of participants, disease, outcome measures, measuring instruments, effects of the interventions on pain intensity, time point assessments and methodological quality.

Another table shows the characteristics of the interventions: author, year, type and brand/model of digital health technologies, interventions, session duration, treatment place, frequency, program duration, supervision and adverse events.

We conducted a narrative synthesis of the findings. This synthesis involved grouping studies based on key themes related to intervention type, delivery modality (e.g., mobile app, web platform, telehealth), and reported effects on pain outcomes. We compared and contrasted study results, highlighting patterns, differences and gaps in the evidence. The narrative approach allowed for a structured summary and interpretation of the findings without statistical pooling.

### 2.5. Methodological Quality of Included Studies

The Downs and Black checklist [33] was used to assess the methodological quality of each study. This checklist consists of 27 items divided into five sections: reporting, external validity, internal validity-bias, internal validity-confounding, and power. All items are scored with 1 point (Yes) or 0 points (No/Unable to determine), except for item five, which is scored with 2 points (Yes), 1 point (Partially) or 0 points (No). Thus, the maximum score on the scale is 28. Based on the results, studies were categorized as: Excellent quality (26–28 points), Good quality (20–25 points), Fair quality (15–19 points), or Poor quality (less than 14 points). Two independent reviewers (OTV and GRS) performed this assessment. To ensure consistency and minimize bias, a third reviewer (ITS) was responsible for reaching consensus in case of any disagreement. The final quality scores were agreed upon by all reviewers.

### 2.6. Risk of Bias Assessment

To assess the risk of bias, we utilized the Cochrane Risk of Bias Assessment Tool [34], which seeks to minimize biases through systematic and explicit methods. This tool assesses bias in seven domains: sequence generation, allocation concealment, blinding of participants and personnel, blinding of outcome assessment, incomplete outcome data, selective outcome reporting, and other bias. Authors assign a judgment of “Low risk”, “High risk” or “Unclear risk” of bias for each domain, justifying their judgements. This evaluation was carried out by two independent reviewers (OTV and GRS). To ensure consistency and minimize bias, a third reviewer (ITS) was responsible for reaching consensus in case of any disagreement. The final scores were agreed upon by all reviewers.

## 3. Results

### 3.1. Search Selection

**Figure 1:**
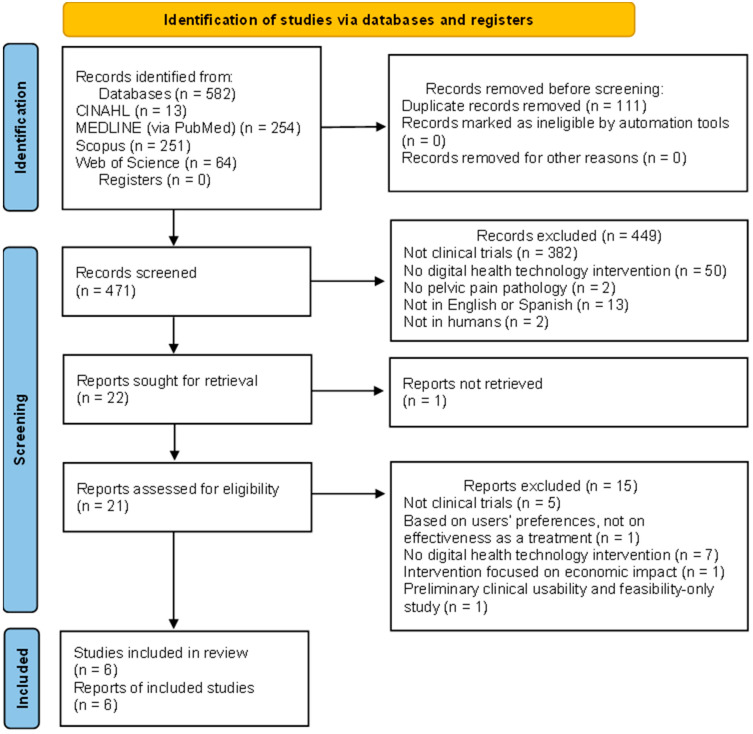
PRISMA flow diagram.

After the initial search, 582 results were retrieved in the four databases mentioned, as shown in Figure 1. Next, 111 duplicates were removed, which left 471 potentially eligible records. After screening by title and abstract, 21 articles remained to be assessed by full-text reading. Of those 21 studies, six randomized clinical trials met the inclusion criteria and were included in the synthesis.

### 3.2. Characteristics of Included Studies

To synthesize the findings, we applied a narrative synthesis approach structured around key features of characteristics of the included studies, the interventions and their reported effects on pain outcomes. Studies were grouped thematically based on the type of digital health technology used (e.g., mobile applications, web-based programs, telehealth platforms), as well as the nature and intensity of the intervention (e.g., duration, frequency, level of supervision).

We systematically compared the effects of interventions on pain intensity across studies, considering the time points of assessment and the tools used to measure pain. Where possible, we identified patterns of effectiveness according to intervention characteristics (e.g., type of technology, delivery setting, or supervision). Divergences in study outcomes were explored in relation to methodological quality, sample size, and variability in intervention parameters. Studies with similar outcomes were discussed together to facilitate comparison, and contradictory findings were noted and contextualized.

This method allowed us to identify consistent trends and highlight areas of heterogeneity in the current evidence base, in the absence of quantitative pooling.

Table 2 shows the characteristics of studies included in this review. Four of them were randomized clinical trials [35,36,37,38], and two were pilot randomized trials [39,40]. The studies used some form of digital health technologies to treat a health condition within the range of diseases included in chronic pelvic pain syndromes. Three of the studies treated endometriosis [35,39,40], two studies treated dysmenorrhea [36,38] and one study treated bladder pain syndrome/interstitial cystitis [37].

The total number of participants was 514, all of them women with a mean age of 29.9 years. The demographic homogeneity of the sample, with all participants being women of a similar mean age, is a typical characteristic of studies on chronic pelvic pain syndromes, given the gendered nature of these conditions. This demographic aspect also limits the generalizability of the findings to other populations. There were two studies conducted in France [35,39], one in Australia [40], one in Germany [36], one in Taiwan [37] and one in China [38].

All studies measured pain intensity. It was measured using an 11-point numerical rating scale in three studies [35,36,39], while the other studies used the visual analog scale [37,40] or the Short-form McGill Pain Questionnaire [38]. The use of different pain measurement tools is common in pain-related studies, but it may also introduce variability in how outcomes are interpreted. The numerical rating scale and visual analog scale are widely used due to their simplicity and sensitivity, but they are limited in their ability to capture the multidimensional nature of pain, which includes affective, sensory, and cognitive components. In contrast, the McGill Pain Questionnaire offers a more comprehensive evaluation, providing richer insights into the pain experience.

Outcome variables were assessed before and after the intervention in all studies. Post-intervention measurements at specific time points were expressed in minutes elapsed since the intervention in two studies [35,39], in hours in one study [40], in weeks in one study [37] and were based on the participants’ menstrual cycles in two studies [36,38].

Regarding the effects of the interventions, this review was focused on whether the pain improved as a result of the interventions. We considered three aspects: pain reduction in the experimental group, pain reduction in the control group, and comparison between pain in the experimental group and pain in the control group after the intervention. In four studies [35,36,37,39], both the experimental group and the control group showed pain reduction from baseline. Yet, only the experimental group’s reduction was statistically significant, with a significant difference in pain intensity between groups at the end of the studies. Apart from these results, one study [40] did not show improvements in pain intensity in any of the groups. In the last study [38], both interventions significantly reduced pain intensity in the experimental and control groups. However, pain reduction between groups was not statistically significant.

Other outcomes related to pain intensity were assessed, such as pain medication intake [35,36], fatigue [35], stress [35], catastrophizing [35], sick leaves [36], body efficiency expectations [36] and quality of life [35,37]. Two studies used disease-specific measuring instruments, such as the O’Leary-Sant symptom and problem indices [37] or the Cox Menstrual Symptom Scale [38]. Finally, only one study measured the occurrence rate of adverse events [38].

### 3.3. Characteristics of Interventions

Characteristics of the interventions in the studies are described in Table 3. Regarding the type of technology used, three studies used virtual reality [34,38,39]; of these, one included a second experimental group (in addition to the virtual reality group) in which telehealth-delivered exercises were used [39]. One study used a mobile phone application [35], one used a combination of internet-based and SMS treatment [36], and one developed and used moxibustion robots [37]. Regarding control treatment, three studies used digital control [34,35,38], two used regular treatments [36,39] and one used traditional manual moxibustion treatment [37].

As for session duration, a single-session treatment was delivered in the two pilot studies [38,39]. In the remaining studies, treatment was applied for a mean of five days a week. One study did not specify an established session duration [36].

Patients were supervised throughout the treatment in two studies [37,38], unsupervised in three studies [34,35,36], and in one study, only patients of one of the two experimental groups were supervised [39]. This is related to the purpose of the interventions of each study. The four studies in which patients were unsupervised were designed for at-home self-management of the disease [34,35,36,39]. Unsupervised interventions, particularly those designed for at-home self-management, are consistent with the growing focus on empowering patients to take control of their own care. This aligns with models of self-management in chronic disease management, where patients are encouraged to monitor their symptoms, make informed decisions, and engage in lifestyle changes outside the clinical setting.

Three studies reported no adverse events related to the intervention [34,36,39]. One study reported mild headache and nausea related to motion sickness from virtual reality [38]. One study reported some adverse events related to the treatment: bruises, deterioration, pain in the hand, pressure pain, shift in the menstruation cycle, dizziness, nausea, pain in the legs and tingling in a finger [35]. One study [37] found a significant difference in the adverse event rate between both groups, with burns being the most reported moderate adverse event. The low incidence of adverse events in most studies supports the safety profile of digital health technologies, which is a critical consideration for their widespread adoption in clinical practice. However, the occurrence of mild adverse events, such as nausea and headaches in virtual reality-based interventions underscores the importance of monitoring patient experiences and adjusting interventions as needed.

### 3.4. Methodological Quality of Studies

The Downs and Black checklist [33] was used to assess the methodological quality of studies included in this review. The total score for each study is shown on Table 1, and the score for each item is summarized in Supplementary Material S3. Of the six articles evaluated, four were classified as good (range: 20–25 points) and two were assessed as fair (range: 15–19 points). The mean score of the studies was 21 (range: 17–25).

### 3.5. Risk of Bias of Studies

**Figure 2:**
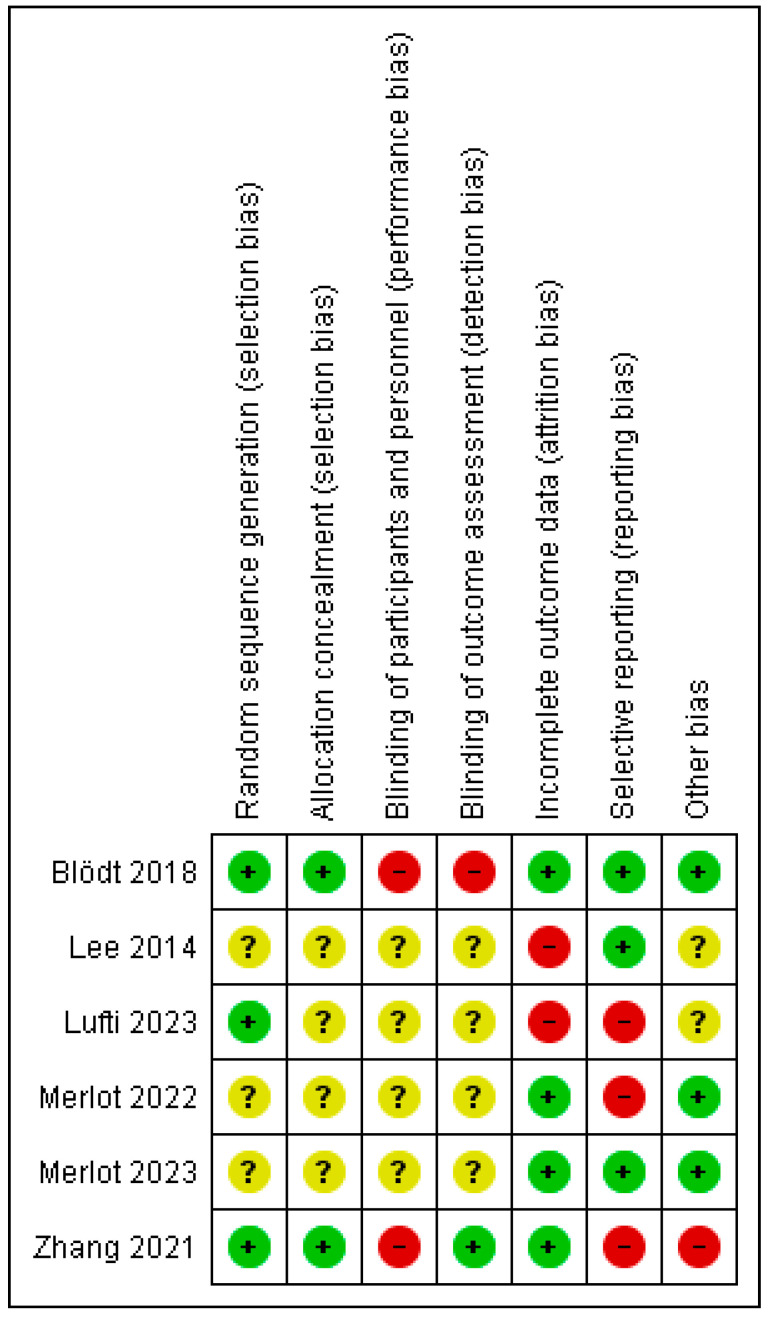
Risk of bias summary [35,36,37,38,39,40].

**Figure 3:**
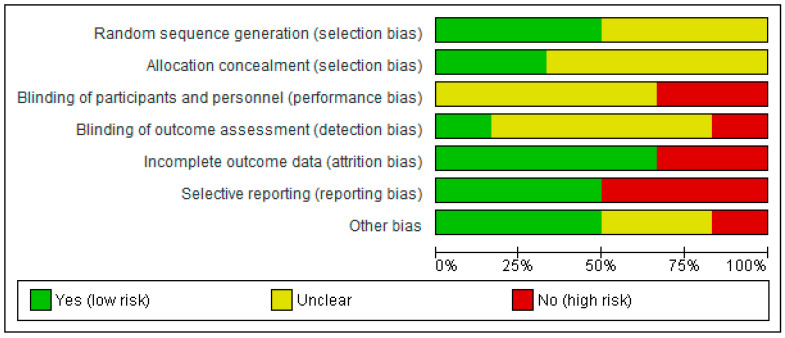
Risk of bias graph.

The Cochrane Risk of Bias Assessment Tool [34] was used to assess the risk of bias of the articles included in this review. Figure 2 and Figure 3 show the summary and the graph of the risk of bias, respectively. Random sequence generation and allocation concealment were evaluated as low risk in three studies [36,38,40] and in two studies [36,38], respectively. These two domains were not assessed as having a high risk of bias in any study. Blinding of participants and personnel was evaluated as high risk in two studies [36,38]. Blinding of outcome measures was assessed as low risk in one study [38] and as high risk in one study [36]. Incomplete outcome data was evaluated as low risk in four studies [35,36,38,39] and as high risk in the other two studies [37,40]. Selective reporting was evaluated as low risk in three studies [35,36,37] and as high risk in the other three studies [38,39,40]. The risk of other bias was assessed as low risk in three studies [35,36,39], as high risk in one study [38] and as unclear risk in two studies [37,40].

## 4. Discussion

### 4.1. Main Findings

The aim of this systematic review was to comprehensively examine the qualitative results of current studies reporting the efficacy of digital health technologies in the treatment of chronic pelvic pain syndromes and to describe the characteristics of these interventions. Six randomized clinical trials were selected and analyzed, showing that digital health significantly reduces pain intensity in the treatment of chronic pelvic pain syndromes.

Most studies reported a statistically significant reduction in pain intensity following the use of digital health interventions, showing a consistent positive trend across different technological approaches.

The digital health interventions included in the review were virtual reality, telehealth, mobile apps, e-health, and robots. The use of digital health technologies in these studies reflects an emerging trend in the management of chronic pelvic pain, which has been recognized for its potential to provide more accessible, scalable, and personalized interventions compared to traditional approaches. digital health technologies offer an innovative means to deliver interventions remotely, thus addressing the challenges of patient engagement and adherence, which are often prevalent in chronic pain conditions.

#### 4.1.1. Virtual Reality

Three studies [35,39,40] in this review used virtual reality to treat chronic pelvic pain syndromes and found that it reduced pelvic and perineal pain and improved quality of life in women with endometriosis, whether in a clinic or in an at-home setting. In both studies by Merlot et al. [35,39], adherence was improved, as patients found it easier to take a pill than to engage in a 20 min virtual reality session; yet, virtual reality had greater benefits than analgesics. A limitation of home use without supervision is that results may be less accurate than in a hospital setting.

In the study by Lutfi et al. [40], virtual reality was found to be as effective as telehealth-delivered exercise, a finding that could guide future research comparing different types of digital health technologies. For example, the review by Lo et al. [41] focuses on the comparison between immersive and non-immersive virtual reality for pain reduction in chronic musculoskeletal conditions. The conclusions support the findings of Merlot et al. [35], who also explored both virtual reality types for treating chronic pelvic pain syndromes. Other studies have shown the potential of virtual reality for the treatment of various chronic pain diseases. An example is the study by Peláez-Vélez et al. [42], in which stroke patients reported improvements in pain, balance, coordination, motor skills, trunk stability and ambulation resulting from virtual reality treatment.

The study by Merlot et al. [35] confirmed the effectiveness and safety of self-repeated administrations of a virtual reality immersive treatment used at home while reducing overall pain medication intake in women diagnosed with endometriosis experiencing moderate-to-severe pelvic pain. The study by Merlot et al. [39] concluded that Endocare, a virtual reality immersive treatment, significantly reduced pain perception compared to a digital control in women living with endometriosis. Interestingly, the effect persisted up to 4 h post-treatment. Finally, the study by Lutfi et al. [40] suggested that a single bout of a “self-managed” virtual reality-delivered exercise may be as efficacious as a single session of “supervised” telehealth-delivered exercise in providing immediate relief from pelvic pain associated with endometriosis.

#### 4.1.2. Other Types of Digital Health

Regarding the use of a mobile app, the clinical trial by Blödt et al. [36] included in this review showed that a mobile app for self-administered acupressure reduced pain and medication use in women with dysmenorrhea.

Moxibustion robots and manual moxibustion were equally effective for treating primary dysmenorrhea, with robots offering a safer procedure, with less risk of adverse events [38]. However, more studies are needed to compare their effectiveness with traditional moxibustion and other pain treatments.

Finally, the study using the E-health system for symptom relief in patients with bladder pain syndrome/interstitial cystitis showed that it was effective in improving quality of life and symptoms, including chronic pelvic pain. This demonstrates that using the internet to educate patients about health is a highly efficient method that can also be applied to other conditions. A study by Lisón et al. [43] showed that the E-health system helped patients improve their health both in the short and long term by using various learning techniques to promote lifestyle changes.

### 4.2. Study Strengths and Limitations

To our knowledge, this review is the first to analyze the effect of various modalities of digital health technologies in chronic pelvic pain syndromes. Its strengths include using the PICOS strategy for inclusion criteria, following PRISMA [31] guidelines, and assessing methodological quality and risk of bias with validated tools such as the Downs and Black [33] scale and the Cochrane Risk of Bias Assessment Tool [34].

The present study has some limitations that need to be addressed, such as the limited number of trials included in the review and the high heterogeneity between studies. This, along with the fact that all the studies had small sample sizes, means that the results of this review should be viewed with caution. Due to the substantial clinical and methodological heterogeneity between the studies, a meta-analysis was not feasible. It is also important to mention that the studies exclusively involved female participants, which may limit applicability to male individuals with chronic pelvic pain syndromes.

The potential gender-specific differences in pain perception, response to digital health interventions, and underlying pathophysiology were not explored. Future research should aim to include more diverse populations, including male participants, to assess the generalizability of digital health technologies for chronic pelvic pain syndromes.

While this manuscript provides a comprehensive overview of various digital health technologies in the management of chronic pelvic pain syndromes, we acknowledge the omission of other significant technologies that hold promise for enhancing patient care in this field. Specifically, emerging technologies such as augmented reality, artificial intelligence, and the internet of things are areas that warrant further exploration. These technologies, while still in early stages of adoption, are exciting future directions that could complement existing digital health technologies in the management of chronic pelvic pain syndromes. Future research should aim to further explore their effectiveness, scalability, and integration into clinical practice to address the various needs of patients with chronic pelvic pain syndromes.

### 4.3. Implications for Clinical Practice

Digital health technologies offer patients the opportunity to self-manage their conditions at home, enhancing their quality of life. This is very important considering the chronic nature of their conditions. The minimal or non-existent adverse events reported highlight the safety of these treatments, making them suitable for home use. As evidence continues to accumulate, digital health interventions could become an integral part of chronic pelvic pain management.

To further strengthen the interpretation of our findings, it is important to consider the potential mechanisms underlying the observed effects of digital health interventions on chronic pelvic pain syndromes. These interventions may reduce pain through various pathways, including distraction and cognitive engagement (e.g., in virtual reality), promotion of self-regulation via biofeedback or guided exercises, and improved adherence through continuous monitoring and personalized feedback. Additionally, digital platforms enhance access to education, behavioral therapies, and support networks, which can modulate pain perception and improve coping strategies. From a practical standpoint, clinicians should consider integrating these technologies as complementary tools to standard care, particularly in patients with limited access to in-person therapies, and select interventions based on individual preferences, digital literacy, and clinical context.

### 4.4. Future Research

Although digital health technologies have been applied in the management of other health conditions for years with good results, their use in the management of chronic pelvic pain syndromes has only started to be studied. More clinical trials with diverse digital health approaches and larger sample sizes are needed to compare their effectiveness in treating chronic pelvic pain syndromes, reduce participant dropouts, and minimize confounding factors. Moreover, research should focus on the long-term impact of these interventions, including their effects on chronic pain-related disability and overall patient quality of life.

## 5. Conclusions

The results of this review suggest that digital health technologies offer some improvement in pain intensity among patients with chronic pelvic pain syndrome. However, the studies showed high heterogeneity, mainly in terms of the type of technology used, which influences the consistency of the results. Virtual reality treatment was the approach with the most solid evidence base due to the number of studies, although more research is needed with larger population samples. This review highlights the need for further studies in this field. Since their adverse events are minimal, digital health technologies may be a good treatment option for patients both in the clinic and at home.

## Figures and Tables

**Table 1:** Details of inclusion and exclusion criteria.

	Inclusion Criteria	Exclusion Criteria
P	Adults with any form of chronic pelvic pain syndromes	
I	Digital health technologies for treatment	
C	Control group, no intervention, intervention without digital health, digital health intervention, placebo, traditional treatment	
O	Pain	
S	RCTs in English, French and Spanish	Not RCTs, protocols, pilots, non-peer-reviewed publications, meeting abstracts or gray literature

RCT: Randomized clinical trials.

**Table 2:** Characteristics of included studies.

Author (Year) [ref]	Country	Sample Size (n)	Age (Mean ± SD)	Disease	Outcome Measures	Measuring Instrument	Effects of the Intervention on Pain Intensity	Time Point Assessment	Methodological Quality
Merlot et al. (2022) [39]	France	45	Total = 32.7 ± 8.02 EG = 32.2 ± 8.02 CG = 33.2 ± 8.12	Endometriosis	Pain intensity	11-point NRS	↓EG * > ↓CG	Pain intensity: baseline/pre-treatment, post-intervention (15 min, 30 min, 45 min, 60 min, 240 min).	22
					Pain relief	5-point CS		Pain relief: post-intervention (15 min, 30 min, 45 min, 60 min, 240 min).	
Merlot et al. (2023) [35]	France	102	Total = 32.9 ± 6.9 EG = 33.7 ± 6.6 CG = 32.1 ± 7.3	Endometriosis	Pain intensity	11-point NRS	↓EG * > ↓CG	Pain intensity: wakeup, pre-treatment, post-intervention (60 min, 120 min, 180 min), bedtime.	21
					Pain relief	5-CS			
					Fatigue	Pichot scale		Pain relief: post-intervention (60 min, 120 min, 180 min).	
					Stress	VAS		Fatigue and stress: wakeup and bedtime.	
					Medication	Report in follow-up diary		Medications: during the study	
					Quality of life	EHP-5		Quality of life: baseline, end of the study	
					Catastrophizing	PCS		Catastrophizing: baseline	
					Global change	PGIC		Global change: end of the study	
					Satisfaction	Global rate		Satisfaction: end of the study	
Lutfi et al. (2023) [40]	Australia	19	EG 1 = 29 ± 7 EG 2 = 27 ± 7 CG = 25 ± 4	Endometriosis	Acute pelvic pain	100 mm VAS	↑EG 1 ≈ ↑EG 2 ↑EG 1 < ↑CG ↑EG 2 < ↑CG	Baseline/pre-treatment	19
								48 h post-intervention	
Blödt et al. (2018) [36]	Germany	221	Total = 24 ± 3.6 EG = 24.4 ± 3.3 CG = 23.7 ± 3.9	Dysmenorrhea	Pain intensity	11-point NRS	↓EG * > ↓CG	Pain intensity: on the days of pain during the 3rd menstrual cycle after therapy starts.	24
					Worst pain intensity during menstruation	NRS			
								Other outcomes: during and after the 1st, 2nd, 3rd and 6th menstrual cycles.	
					Duration of pain	Number of days with pain			
					Responder rates	50% reduction in mean pain intensity on the days with pain compared to the corresponding baseline value			
					Pain medication	Number of days with pain medication			
					Sick leave	Days of absence from work or school			
					Body efficiency expectations	Body efficacy expectation 5 item scale			
Lee et al. (2014) [37]	Taiwan	65	EG = 46.5 ± 10.2 CG = 49.5 ± 11.8	BPS/IC	Pain and urgency	VAS	↓EG * > ↓CG	Baseline/pre-treatment	18
								8 weeks post-intervention	
					Disease severity	O’Leary-Sant symptom and problem indices			
					Quality of life	SF-36			
Zhang et al. (2021) [38]	China	62	EG = 23.1 ± 2.8 CG = 22.6 ± 3.8	PD	Pain degree	SF-MPQ	↓EG * ≈ ↓CG *	Baseline	24
					Symptoms of PD	CMSS			
					AEs	Occurrence rate of AEs (cases with AEs/the total number of cases × 100%)		3rd menstrual cycle post-intervention	
								6th menstrual cycle post-intervention	

Abbreviations: AEs: Adverse events; BPS/IC: Bladder pain syndrome/interstitial cystitis; CG: Control group; CMSS: Cox Menstrual Symptom Scale; CS: Categorical scale; EG: Experimental group; EHP-5: Endometriosis health profile; NRS: Numerical rating scale; NSAIDs: Non-steroidal anti-inflammatory drugs; PCS: Pain Catastrophizing Scale; PD: Primary dysmenorrhea; PGIC: Patient’s global impression of change; RCT: Randomized clinical trial; SD: Standard deviation; SF-36: 36-Item Short-form Health Survey; SF-MPQ: Short-form McGill Pain Questionnaire; VAS: Visual analog scale. ↓: pain decreased between baseline and post-treatment measures in the group. ↑: pain increased between baseline and post-treatment measures in the group. *: difference between baseline and post-treatment measures is statistically significant in the same group. ≈: difference between baseline and post-treatment measures is approximately the same. **bold:** difference is statistically significant between groups.

**Table 3:** Characteristics of interventions.

Author (Year) [ref]	Type of Digital Health Technology	Brand/Model of Digital Health Technology	Interventions	Session Duration	Treatment Place	Frequency	Program Duration	Supervision	Adverse Events
Merlot et al. (2022) [39]	VR	In EG: Software: “Endocare” Class I medical software. Hardware: VR headset (Oculus Quest) with high-quality headphones (APK K-240-MKII)	EG: Endocare software offers treatment consisting of a combination of auditory (e.g., alpha/theta binaural beats, nature-based sounds) and visual (e.g., bilateral alternative stimulations consisting of a sphere appearing and moving on a horizontal axis) therapeutic procedures integrated in a 3D VR environment.	20 min	IFEMEndo, a clinic that specializes in endometriosis	Single session	Single session	Through the entire treatment	7 (15%) mild-to-moderate cases. 4 (8%) probably unrelated cases. 3 (6%) possibly related to the Endocare treatment, described as a mild headache and nausea related to motion sickness.
			CG: Digital control program displayed through a tablet (Samsung Galaxy Tab A) with same high-quality headphones. Same context, environment and duration as the Endocare treatment but without any immersive effects, nor the auditory and visual stimuli. A soundtrack composed of nature sounds related to the projected image.						
Merlot et al. (2023) [35]	VR	In EG and CG: Software: “Endocare” Class I medical software. Hardware: Oculus Quest 2 VR headsets and AKG K-240MKII audio headphones	EG: Treatment combining auditory and visual therapeutic stimulations integrated in a 3D VR environment, including binaural beats, verbal hypnotic injunction, nature-based sounds, distraction of attention, and bilateral alternative stimulations.	20 min	At home	Up to twice a day (minimum of 3 h between exposure) for at least 2 days and up to 5 days from the 1st day of painful menstruation, of de next menstrual cycle after starting the study.	5 days	No supervision.	None
			CG: Same hardware as EG, 20 min audio-video composition similar to Endocare (same context, environment, and duration) with exposure to nature sounds, but without Endocare’s stimulations.						
Lutfi et al. (2023) [40]	VR and telehealth	In EG1: the exercise apps were: Dance Central, Beat Saber, The Thrill of the Fight, Space Pirate Trainer, Fruit Ninja, OhShape, Racket NX, Table Tennis VR, Racket Fury, Swords of Gargantua, BoxVR, Superhot VR, VZ Fit Play, and VZFit Explorer.	EG1 (VR exercises): A 10 min VR pain-distraction experience using a list of apps previously shown to reduce pain; and 50 min of exercise using one of the applications based on participants’ preferences and goals. EG2 (telehealth delivered exercises): The exercises included cardiorespiratory exercises and stretching and specific stabilization exercises of the muscles of the lumbopelvic area.	60 min	Not specified	Single session	Single session	EG1: supervised EG2: unsupervised	None
			CG: Were instructed to continue with their activities of daily living.						
Blödt et al. (2018) [36]	Mobile phone app	In EG and CG: Smartphone app AKUD (Software development: Smart Mobile Factory, Berlin, Germany)	EG: AKUD app with access to self-acupressure specific features: explanations of the acupressure procedure, drawings, videos and photos of the acupressure points and a timer to guide the 1 min acupressure of each point. The acupressure points SP6, LI4 and LR3 were used on both sides.	6 min (1 min per point)	At home via smartphone	5 days before period: at least once a day, twice if possible. During menstruation, on painful days: at least twice a day, up to 5 times.	6 menstrual cycles (6 months)	No supervision	EG: 15 patients reported at least 1 suspected AEs: bruises (n = 5), deterioration (n = 3), pain in the hand (n = 1), pressure pain (n = 1), shift in menstruation cycle (n = 3), dizziness (n = 1), nausea (n = 1), pain in the legs (n = 1), tingling in a finger (n = 2). Two serious AEs occurred in each group. EG: hip surgery, hospitalization due to dizziness. CG: surgery of the nose, appendix surgery. None was considered related to the trial or the trial intervention.
			CG: AKUD app without acupressure specific features, access to just menstrual cycle visualization, questionnaires and diary.						
Lee et al. (2014) [37]	E-Health (Internet and SMS)	In EG: Web service installed in the web server to respond to or communicate with the mobile phone by sending/receiving short messages through the Hinet message center.	EG: Health education through web service, for consolidating healthy dieting habits and lifestyle. Health education questions to check their compliance in following the suggestions of the provided educational materials. SMS server to handle the cases of flare symptoms + regular treatments.	No session duration, at-home self-management	At home via smartphone or through an Internet browser	Weekly	8 weeks	No supervision	None
			CG: Regular treatments.						
Zhang et al. (2021) [38]	Robots	In EG: MR composed of a 6-degree-of-freedom robot arm, a controller, an infrared temperature sensor (MLX90614ESF), a laser ranging sensor (ATK-VL530L0X), and a moxa stick propulsion device.	EG: Moxibustion treatment through a robot that monitors and adjusts the skin temperature of CV4 point and the distance along with the change in skin temperature.	30 min	In the Acupuncture Department of Affiliated Hospital of Chengdu University of Traditional Chinese Medicine.	Once a day, 5 days a week. Starting 5 days before the beginning of menses until forthcoming menstruation	3 menstrual cycles of treatment and 3 menstrual cycles of follow-up	Through the entire treatment	Total number of AEs: 37 cases (in 815 patients). Grading: 31 mild cases, 6 moderate cases, no severe cases. AEs in EG: 9 cases. Rate of 2.1% (9 in 424 sessions). Cases: itching (4), bowel changes (3), menstrual changes (1), menorrhagia (1). AEs in CG: 28 cases. Rate of 7.2% (28 in 391 sessions). Cases: first grade burns (2), second grade burns (4), itching (9), bowel changes (7), menstrual changes (3), menorrhagia (2), fatigue (1).
			CG: Traditional manual moxibustion treatment.						

Abbreviations: AEs: Adverse effects; CG: Control group; CV-4: Guanyuan acupoint; EG: Experimental group; LI4: Hegu acupoint; LR3: Taichong acupoint; MR: Moxibustion robot; SP6: Sanyinjiao acupoint; VR: Virtual reality.

## Data Availability

Data and other materials are available from the corresponding author.

## Supplementary Materials

The following supporting information can be downloaded at: https://www.mdpi.com/article/10.3390/healthcare13212665/s1.
