# Supplementary material for: Digital Health Technologies in the Treatment of Chronic Pelvic Pain Syndromes: A Systematic Review of Randomized Clinical Trials
Source: Healthcare (Basel). 2025 Oct 22;13(21):2665. doi: 10.3390/healthcare13212665 (PMC12608623; doi:10.3390/healthcare13212665)
Supplement: Supplementary file 1 [file healthcare-13-02665-s001.zip › healthcare-3867355-supplementary.pdf]

Supplementary material S1. Search strategy.

Database	CINAHL
Date	10/07/2024
Strategy	#1 AND #2 AND #3
#1	AB (“Pelvic Pain”[MeSH] OR “chronic pelvic pain”)
#2	AB (“prostate” OR “Prostatitis”[MeSH] OR “bladder” OR “scrotal” OR “testicular” OR “epididymal” OR “penile” OR “urethral” OR “post-vasectomy” OR “vulvar” OR “vestibular” OR “clitorial” OR “endometriosis” OR “CPPPS” OR “dysmenorrhea” OR “irritable bowel” OR “chronic anal” OR “intermittent chronic anal” OR “pudendal pain syndrome” OR “Dyspareunia”[MeSH] OR “sexual dysfunction” OR “pelvic organ” OR “pelvic floor muscle” OR “abdominal muscle” OR “spinal” OR “coccyx” OR “hip muscle” OR “chronic post-surgical pain syndrome”)
#3	AB (“Video Games”[MeSH] OR “Game*” OR “Gaming” OR “gamification” OR “Exergaming”[MeSH] OR “exergam*” OR “Wii” OR “Nintendo” OR “Kinect” OR “Xbox” OR “PlayStation” OR “virtual” OR “Virtual Reality”[MeSH] OR “Virtual Reality Exposure Therapy”[MeSH] OR “computer-based” OR “Steam” OR “Mobile Applications”[MeSH] OR “app” OR “app-based” OR “Digital Health”[MeSH] OR “mHealth” OR “eHealth” OR “Telemedicine”[MeSH] OR “technologies” OR “artificial intelligence” OR “telehealth” OR “biomedical technology”[MeSH] OR “medical informatics applications”[MeSH] OR “smartphone” OR “mobile*” OR “computer” OR “wearable devices”)
**Database**	**MEDLINE (via Pubmed)**
Date	10/07/2024
Strategy	#1 AND #2 AND #3
#1	(“Pelvic Pain”[MeSH] OR “chronic pelvic pain”)
#2	(“prostate” OR “Prostatitis”[MeSH] OR “bladder” OR “scrotal” OR “testicular” OR “epididymal” OR “penile” OR “urethral” OR “post-vasectomy” OR “vulvar” OR “vestibular” OR “clitorial” OR “endometriosis” OR “CPPPS” OR “dysmenorrhea” OR “irritable bowel” OR “chronic anal” OR “intermittent chronic anal” OR “pudendal pain syndrome” OR “Dyspareunia”[MeSH] OR “sexual dysfunction” OR “pelvic organ” OR “pelvic floor muscle” OR “abdominal muscle” OR “spinal” OR “coccyx” OR “hip muscle” OR “chronic post-surgical pain syndrome”)
#3	(“Video Games”[MeSH] OR “Game*” OR “Gaming” OR “gamification” OR “Exergaming”[MeSH] OR “exergam*” OR “Wii” OR “Nintendo” OR “Kinect” OR “Xbox” OR “PlayStation” OR “virtual” OR “Virtual Reality”[MeSH] OR “Virtual Reality Exposure Therapy”[MeSH] OR “computer-based” OR “Steam” OR “Mobile Applications”[MeSH] OR “app” OR “app-based” OR “Digital Health”[MeSH] OR “mHealth” OR “eHealth” OR “Telemedicine”[MeSH] OR “technologies” OR “artificial intelligence” OR “telehealth” OR “biomedical technology”[MeSH] OR “medical informatics applications”[MeSH] OR “smartphone” OR “mobile*” OR “computer” OR “wearable devices”)
**Database**	**Scopus**
Date	10/07/2024
Strategy	#1 AND #2 AND #3
#1	title-abs-key (“chronic pelvic pain”)
#2	title-abs-key (“prostate” OR “bladder” OR “scrotal” OR “testicular” OR “epididymal” OR “penile” OR “urethral” OR “post-vasectomy” OR “vulvar” OR “vestibular” OR “clitorial” OR “endometriosis” OR “CPPPS” OR “dysmenorrhea” OR “irritable bowel” OR “chronic anal” OR “intermittent chronic anal” OR “pudendal pain syndrome” OR “dyspareunia” OR “sexual dysfunction” OR “pelvic organ” OR “pelvic floor muscle” OR “abdominal muscle” OR “spinal” OR “coccyx” OR “hip muscle” OR “chronic post-surgical pain syndrome”)
#3	title-abs-key (“Game*” OR “Gaming” OR “gamification” OR “exergam*” OR “Wii” OR “Nintendo” OR “Kinect” OR “Xbox” OR “PlayStation” OR “virtual” OR “computer-based” OR “Steam” OR “app” OR “app-based” OR “Digital Health” OR “mHealth” OR “eHealth” OR “Telemedicine” OR “technologies” OR “artificial intelligence” OR “telehealth” OR “biomedical technology” OR “medical

	informatics applications" OR "smartphone" OR "mobile*" OR "computer" OR "wearable devices")
**Database**	**Web of Science**
Date	10/07/2024
Strategy	#1 AND #2 AND #3
#1	TS = ("Pelvic Pain"[MeSH] OR "chronic pelvic pain")
#2	TS = ("prostate" OR "Prostatitis"[MeSH] OR "bladder" OR "scrotal" OR "testicular" OR "epididymal" OR "penile" OR "urethral" OR "post-vasectomy" OR "vulvar" OR "vestibular" OR "clitorial" OR "endometriosis" OR "CPPPS" OR "dysmenorrhea" OR "irritable bowel" OR "chronic anal" OR "intermittent chronic anal" OR "pudendal pain syndrome" OR "Dyspareunia"[MeSH] OR "sexual dysfunction" OR "pelvic organ" OR "pelvic floor muscle" OR "abdominal muscle" OR "spinal" OR "coccyx" OR "hip muscle" OR "chronic post-surgical pain syndrome")
#3	TS = ("Video Games"[MeSH] OR "Game*" OR "Gaming" OR "gamification" OR "Exergaming"[MeSH] OR "exergam*" OR "Wii" OR "Nintendo" OR "Kinect" OR "Xbox" OR "PlayStation" OR "virtual" OR "Virtual Reality"[MeSH] OR "Virtual Reality Exposure Therapy"[MeSH] OR "computer-based" OR "Steam" OR "Mobile Applications"[MeSH] OR "app" OR "app-based" OR "Digital Health"[MeSH] OR "mHealth" OR "eHealth" OR "Telemedicine"[MeSH] OR "technologies" OR "artificial intelligence" OR "telehealth" OR "biomedical technology"[MeSH] OR "medical informatics applications"[MeSH] OR "smartphone" OR "mobile*" OR "computer" OR "wearable devices")

Supplementary material S2. Excluded studies in the last screening with reasons for exclusion (n =15).

Reference	Reason for exclusion
Rodrigues, Y. T., Silva, T. C. L. A., Radytė, E., Bernatavičius, E., Cook, A. A., Carvalho, M. L. A. S., Macedo, L. E. S., Oliveira, J. M. P., Martins, T. D., Fonseca, M. E., Micussi, M. T. A. B. C., & Pegado, R. (2024). Clinical usability study of a home-based self-administration transcranial direct current stimulation for primary dysmenorrhea: A randomized controlled trial. PloS one, 19(5), e0301851. https://doi.org/10.1371/journal.pone.0301851	Preliminary clinical usability and feasibility-only study.
Valedi, S., MoradiBaglooei, M., Ranjbaran, M., Chegini, V., Griffiths, M. D., & Alimoradi, Z. (2022). The efficacy of eye movement desensitization and reprocessing in reducing anxiety among female university students with primary dysmenorrhea. BMC psychology, 10(1), 50. https://doi.org/10.1186/s40359-022-00757-0	Intervention does not meet the digital health technology definition
Miazga, E., Starkman, H., Schroeder, N., Nensi, A., & McCaffrey, C. (2024). Virtual Mindfulness-Based Therapy for the Management of Endometriosis Chronic Pelvic Pain: A Novel Delivery Platform to Increase Access to Care. Journal of obstetrics and gynaecology Canada: JOGC = Journal d'obstetrique et gynecologie du Canada: JOGC, 46(6), 102457. Advance online publication. https://doi.org/10.1016/j.jogc.2024.102457	It is not a clinical trial
Merhi, Z., Emdin, D., Bosman, L., Incledon, T., & Smith, A. H. (2023). Ozone Sauna Therapy (OST) and Pulsed Electromagnetic Field Therapy (PEMF) delivered via the HOCATT machine could improve endometriosis pain along with lowering serum inflammatory markers. American journal of reproductive immunology (New York, N.Y. : 1989), 89(4), e13690. https://doi.org/10.1111/aji.13690	It is not a clinical trial
Song, M., & Kanaoka, H. (2018). Effectiveness of mobile application for menstrual management of working women in Japan: randomized controlled trial and medical economic evaluation. Journal of medical economics, 21(11), 1131–1138. https://doi.org/10.1080/13696998.2018.1515082	Intervention focused on economic impact
Yu, S., Wen, Y., Xia, W., Yang, M., Lv, Z., Li, X., Li, W., Yang, S., Hu, Y., Liang, F., & Yang, J. (2018). Acupoint herbal plaster for patients with primary dysmenorrhea: study protocol for a randomized controlled trial. Trials, 19(1), 348. https://doi.org/10.1186/s13063-018-2682-8	Intervention does not meet the digital health technology definition
Blödt, S., Schützler, L., Huang, W., Pach, D., Brinkhaus, B., Hummelsberger, J., Kirschbaum, B., Kuhlmann, K., Lao, L., Liang, F., Mietzner, A., Mittring, N., Müller, S., Paul, A., Pimpao-Niederle, C., Roll, S., Wu, H., Zhu, J., & Witt, C. M. (2013). Effectiveness of additional self-care acupressure for women with menstrual pain compared to usual care alone: using stakeholder engagement to design a pragmatic randomized trial and study protocol. Trials, 14, 99. https://doi.org/10.1186/1745-6215-14-99	It is not a clinical trial
Hosono, T., Takashima, Y., Morita, Y., Nishimura, Y., Sugita, Y., Isami, C., Sakamoto, I., Tagami, K., Hidaka, Y., & Suzuki, A. (2010). Effects of a heat- and steam-generating sheet on relieving symptoms of primary dysmenorrhea in young women. The journal of obstetrics and gynaecology research, 36(4), 818–824. https://doi.org/10.1111/j.1447-0756.2010.01237.x	Intervention does not meet the digital health technology definition
Morin, M., Dumoulin, C., Bergeron, S., Mayrand, M. H., Khalifé, S., Waddell, G., Dubois, M. F., & PVD Study Group (2021). Multimodal physical therapy versus topical lidocaine for provoked vestibulodynia: a multicenter, randomized trial. American journal	Intervention does not meet the digital health technology definition

of obstetrics and gynecology, 224(2), 189.e1–189.e12. https://doi.org/10.1016/j.ajog.2020.08.038	
Berman, S. M., Naliboff, B. D., Suyenobu, B., Labus, J. S., Stains, J., Ohning, G., Kilpatrick, L., Bueller, J. A., Ruby, K., Jarcho, J., & Mayer, E. A. (2008). Reduced brainstem inhibition during anticipated pelvic visceral pain correlates with enhanced brain response to the visceral stimulus in women with irritable bowel syndrome. The Journal of neuroscience: the official journal of the Society for Neuroscience, 28(2), 349–359. https://doi.org/10.1523/JNEUROSCI.2500-07.2008	It is not a clinical trial
Salsi, B., Ganassi, G., Lopopolo, G., Callarelli, S., Comito, A., Fusco, I., & Isaza, P. G. (2023). Approach of Chronic Pelvic Pain with Top Flat Magnetic Stimulation. Advances In Urology, 2023, 1-7. https://doi.org/10.1155/2023/9983301	It is not a clinical trial
Anderson, R. U., Wise, D., Sawyer, T., Nathanson, B. H., & Smith, J. N. (2015). Equal Improvement in Men and Women in the Treatment of Urologic Chronic Pelvic Pain Syndrome Using a Multi-modal Protocol with an Internal Myofascial Trigger Point Wand. Applied Psychophysiology and Biofeedback, 41(2), 215-224. https://doi.org/10.1007/s10484-015-9325-6	Intervention does not meet the digital health technology definition
Lee, J., & Kim, J. (2019). Can menstrual health apps selected based on users' needs change health-related factors? A double-blind randomized controlled trial. Journal of the American Medical Informatics Association: JAMIA, 26(7), 655–666. https://doi.org/10.1093/jamia/ocz019	The aim of the study is to improve the functionalities of menstrual apps based on users' preferences, not on their effectiveness as a treatment
Lee, J. M., & Kim, K. H. (2017). Effect of near-infrared rays on female menstrual pain in Korea. Nursing & health sciences, 19(3), 366–372. https://doi.org/10.1111/nhs.12356	Intervention does not meet the digital health technology definition
Hong, G. Y., Shin, B. C., Park, S. N., Gu, Y. H., Kim, N. G., Park, K. J., Kim, S. Y., & Shin, Y. I. (2016). Randomized controlled trial of the efficacy and safety of self-adhesive low-level light therapy in women with primary dysmenorrhea. International journal of gynaecology and obstetrics: the official organ of the International Federation of Gynaecology and Obstetrics, 133(1), 37–42. https://doi.org/10.1016/j.ijgo.2015.08.004	Intervention does not meet the digital health technology definition

Supplementary material S3. Methodological quality of included studies.

	Study quality										External validity			Study bias							Confounding and selection bias						Study power		
**Author (year)[ref]**	**1**	**2**	**3**	**4**	**5**	**6**	**7**	**8**	**9**	**10**	**11**	**12**	**13**	**14**	**15**	**16**	**17**	**18**	**19**	**20**	**21**	**22**	**23**	**24**	**25**	**26**	**27**	**TOTAL**	**QUALITY**
Merlot et al. (2022) [illegible]	1	1	1	1	2	1	1	1	1	1	1	1	0	0	0	1	1	1	1	1	1	0	1	0	0	1	1	22	GOOD
Merlot et al. (2023) [illegible]	1	1	1	1	2	1	1	1	1	1	1	1	0	0	0	1	1	1	1	1	0	0	1	0	0	1	1	21	GOOD
Lutfi et al. (2023) [illegible]	1	1	1	1	2	1	1	1	1	1	0	1	1	0	0	1	1	1	1	0	0	1	1	0	0	0	0	19	FAIR
Blödt et al. (2018) [illegible]	1	1	1	1	1	1	1	1	1	1	1	1	1	0	0	1	1	1	1	1	1	1	1	0	1	1	1	24	GOOD
Lee et al. (2014) [illegible]	1	1	0	1	1	1	1	0	1	1	0	1	0	0	0	1	1	1	1	1	1	0	1	0	0	1	1	18	FAIR
Zhang et al. (2021 [illegible]	1	1	1	1	2	1	1	1	1	0	1	1	0	0	1	1	1	1	1	1	1	1	1	1	0	1	1	24	GOOD
